# International multicenter tetralogy of Fallot registry: identifying predictors of adverse outcomes using cardiac MRI parameters

**DOI:** 10.1186/1532-429X-13-S1-P187

**Published:** 2011-02-02

**Authors:** Anne Marie Valente, Sonya Babu-Narayan, Gabriele Egidy Assenza, Sarah P Evans, Kimberlee Gauvreau, Maarten Groenink, Ryo Inuzuka, Philip Kilner, Zeliha Koyak, Michael J Landzberg, Barbara Mulder, Michael A Gatzoulis, Andrew J Powell, Rachel Wald, Tal Geva

**Affiliations:** 1 Children's Hospital Boston, Boston, MA, USA; 2 Royal Brompton Hospital and Imperial College, London, UK; 3 Academic Medical Centre, Amsterdam, Netherlands; 4 Toronto General Hospital,Toronto Congenital Cardiac Centre for Adults, Toronto, ON, Canada

## Introduction

Although survival of patients with repaired tetralogy of Fallot (TOF) into adulthood exceeds 90%, hemodynamic and electrophysiologic abnormalities contribute to substantial morbidity in this population. The rate of late complications such as exercise intolerance, heart failure, tachyarrhythmias, and death accelerates in the third decade of life. However, identifying predictors for adverse outcomes remains difficult, as the event rates for major outcomes, such as ventricular tachycardia (VT) and death are low. Small, single center studies have suggested that cardiac magnetic resonance (CMR) measures of ventricular size and function are independent predictors of major adverse clinical outcomes. However, the small number of outcome events and the single center study design limit general acceptance of these findings. A multicenter registry may allow identification of generalizable predictors of major adverse outcomes and will provide opportunities for robust analyses to address clinically relevant questions in this population.

## Purpose

The purpose of this report is to describe the creation of an international multicenter registry to identify CMR predictors of adverse outcomes in patients with repaired TOF.

## Methods

Four large congenital heart centers enrolled subjects with repaired TOF into a prospectively designed database with a statistical and a CMR core. Demographic and clinical information, QRS duration, exercise, Holter monitor, interventional procedures, and outcome data were collected. CMR images were transferred to the core laboratory where ventricular volumes, mass and flow data were contoured by investigators blinded to patient outcome.

## Results

Of the 1142 patients enrolled to date (median age 28 years, 54% male), 42% had a palliative shunt prior to TOF repair, 454 (40%) had undergone pulmonary valve replacement, and 236 (21%) had documented arrhythmia (sustained VT in 29 subjects). At the time of data collection, 21 patients (2%) were deceased. Of the 1490 CMR examinations received to date, 1262 have been analyzed by the core laboratory and results will be presented.

## Conclusions

This multicenter registry will allow much greater statistical power to evaluate major outcomes in patients with repaired TOF. The initial release of this registry includes 4 large congenital heart centers, but ultimately it will transition to a longitudinal registry with involvement of additional centers. The process of establishing a core congenital CMR laboratory for uniform data analysis and an electronic database for standardized data entry are the first steps to identifying predictors of adverse outcomes meaningful to this patient population.

